# Task-irrelevant object response to action enhances the sense of agency for controlling the object in automation

**DOI:** 10.1038/s41598-022-20125-7

**Published:** 2022-09-22

**Authors:** Ryoichi Nakashima, Takatsune Kumada

**Affiliations:** 1 Department of Intelligence Science and Technology, Graduate School of Informatics, Kyoto University, Yoshida-Honmachi, Sakyo-Ku, Kyoto, 606-8501 Japan; 2 RIKEN CBS-TOYOTA Collaboration Center, RIKEN, 2271-130 Anagahora, Shimoshidami, Moriyama-Ku, Nagoya, Aichi 463-0003 Japan

**Keywords:** Human behaviour, Psychology

## Abstract

The sense of agency (SoA) refers to the experience of controlling our bodies and tools. Recent automated systems require the operators to have less manual control, which decreases the SoA. This study investigated how to increase the SoA when operating automated systems, by focusing on the effect of an object’s responses to operators’ actions on the SoA. Participants applied brakes to a moving black circle by pressing a key, in order to stop the circle near a goal. Then, they estimated their SoA for stopping the circle. We informed them that there were automatic control trials in which the circle stopped independently of their keypress (86% of the trials). The circle’s color briefly changed to white (i.e., flashed) when they pressed the key in a half of the automatic control trials. The SoA was higher with the flash than without it. However, the SoA neither increased when the circle flashed independently of a keypress nor when another object flashed due to a keypress. Furthermore, the keypress contingent object-flash did not influence the SoA when the participants controlled the circle manually. These results indicated that spatiotemporally contingent object responses to actions can enhance the SoA in automatic control situations.

## Introduction

The sense of agency (SoA) refers to the subjective experience of controlling the activities of our body and external events^1–4^. Many studies, including psychology, neuroscience, and engineering, have investigated the SoA. Psychology and neuroscience researchers have examined the mechanisms underlying the SoA. The engineering researchers have been interested in applied aspects of the SoA which are closely related to rapidly developing human–machine interaction technology. It is essential to clarify how people perceive the SoA during human–machine interactions and collaborative control of objects/tools with a system.

One critical characteristic of modern technology is the development of automation systems requiring reduced manual control by operators. Operators using automation technologies feel that such technologies are easy to operate because they need not operate them alone. However, they might simultaneously feel less engaged in the operation of these technologies due to the lack of SoA. Berberian et al.^5^ demonstrated that SoA decreased when the automation level increased from completely manual control to completely automatic control.

The SoA is critical for human behavior and mental condition. For example, the SoA is related to action selection^6–8^, and essential for skill-learning^9^. Moreover, self-generated motion, i.e., motion with an SoA, could be processed more efficiently than motion without it^10,11^, and could modulate visual perception^12–14^. Thus, automation might change operators’ behavior, visual processing and so on by decreasing the SoA.

More importantly, serious problems could arise during automation if the source of control (humans or machines) is ambiguous and the human is “out-of-the-loop” of the control^15,16^. The “out-of-the-loop” performance problem impairs the ability of automatic system operators to take over manual operations in the event of automation failure. Even operators in modern fully automatic control systems sometimes have to take control because current systems cannot manage all possible problems, particularly dangerous or complex ones. Therefore, the “out-of-the-loop” performance problem has potentially severe consequences in many automation situations.

To prevent “out-of-the-loop” performance problems, a system’s operators must be involved in controlling the system. One possible method to accomplish this is maintaining the operators’ SoA during automatic control situations^17^. If operators feel an SoA for controlling an object, they should feel involved in the control loop. That is, feeling the SoA could be the basis for being involved in automated systems’ control loop in many automation situations.

Recent studies have investigated the SoA when conducting activities with the system assistance^18–20^. These studies showed that improving the operators’ performance in tasks by system assistance could enhance the SoA. For example, participants in an experiment by Wen et al.^20^ controlled a moving circle continuously and led the circle to a goal. In some trials, a computer helped participants improve their performance through computer-assisted control (in these trials, participants’ operation was not fully reflected in the output). The results showed that the SoA was higher in the computer-assisted than in the self-control condition. Interestingly, the SoA increased through assistance even if the operators explicitly recognized that a computer was assisting them in conducting the task^18^. These studies also demonstrated increasing SoA resulting from computer-assisted control when there were long delays between participants’ actions and outcomes, which makes the action-outcome relationship uncertain. This finding implies that the SoA could be maintained when actions and outcomes are not directly related but operators’ intentions and the automation systems’ outcomes match. Consistent with this implication, Nakashima and Kumada^21^ suggested that the SoA decrement during automation can be attenuated by the tool-use situation’s characteristics, including the presence of goals and the gradual emergence of outcomes. The former might make operators feel that their intentions and outcomes of the system match, and the latter can cause an uncertain relationship between actions and outcomes. These characteristics make operators feel that the action and outcome are illusorily matched.

The present study examined whether the SoA increases in automatic control situations with a goal and a gradually emerging outcome, in order to clarify the SoA during human–machine interactions further. We specifically focused on the effect of task-irrelevant object responses on the SoA for controlling an object. The SoA in automatic control situations might result from illusorily binding between the operators’ actions and outcomes generated by the system. Specific responses of an object to operators’ actions might make operators feel that they control the object due to the illusorily binding the action and outcome.

## Experiment 1

**Figure 1:**
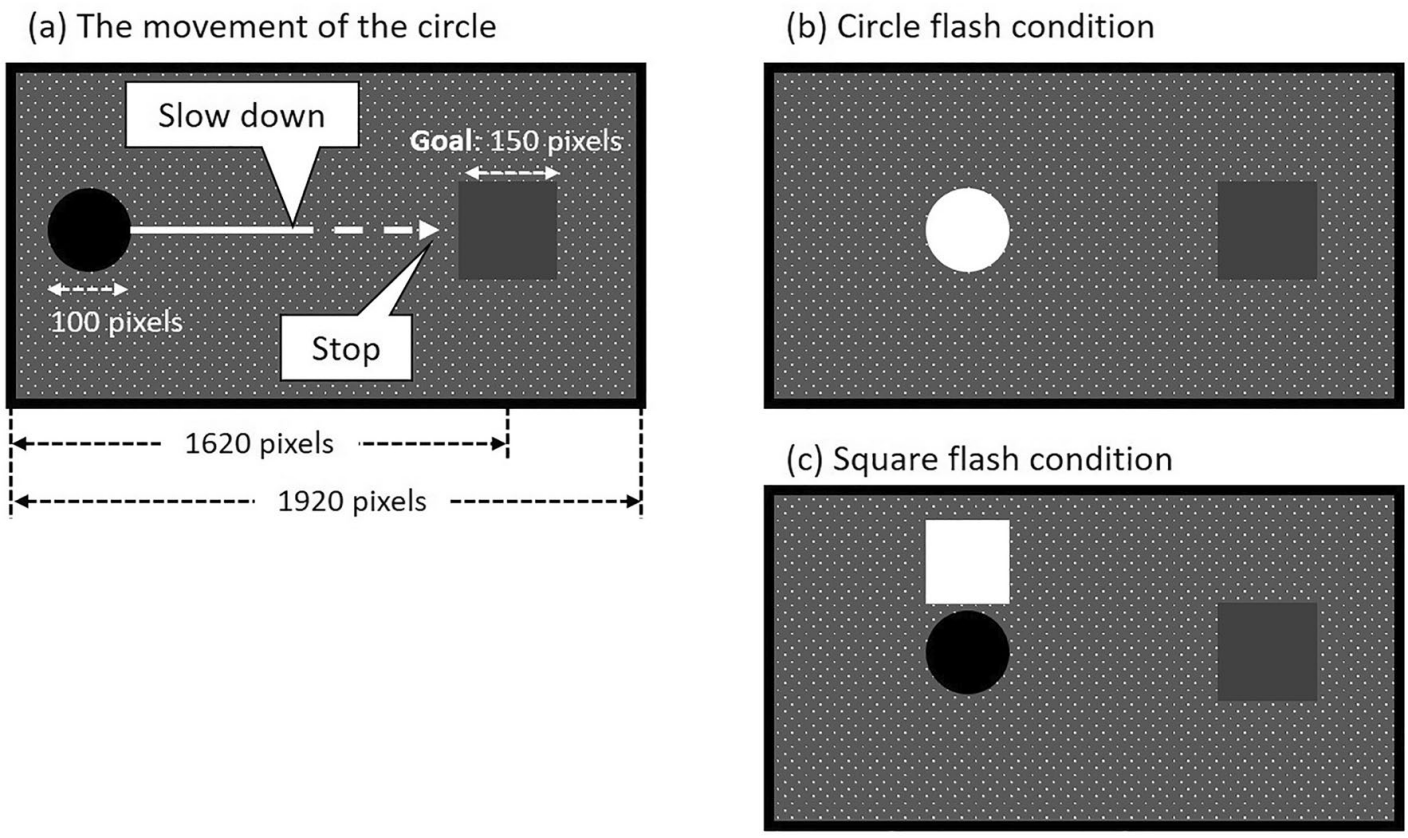
Experimental displays’ examples. (**a**) The circle’s movement: When the brake is applied, the circle slows down and stops. (**b**) The circle flashing in Experiments 1 and 2. In Experiment 1, the circle flashed when participants pressed the key in the action-contingent circle flash block, and at random in the random circle flash block. (**c**) The square flashing in Experiment 2 (for details, see Experiment 2). In Experiment 2, when participants pressed the key, the circle itself flashed or the white square appeared briefly above the circle.

The purpose of Experiment 1 was to examine the effect of a task-irrelevant object response on the operator’s SoA for controlling the object which was controlled by an automatic control system. The task in this experiment was the goal-present and gradual-stop situation introduced by Nakashima and Kumada^21^, where participants were asked to stop a moving circle near a specific goal by pressing a key as a brake, which slowed and stopped the circle (see Fig. 1a). Importantly, we explicitly informed the participants that the system often controlled the circle by stopping the circle near the goal area independently of their keypresses in the experimental trials.

We defined object flashes associated with the participants’ keypresses as the object task-irrelevant responses. We examined whether flashing an object in response to participants’ keypresses enhanced the participants’ SoA for stopping the object even though the object was automatically controlled (Fig. 1b). In Experiment 1’s “action-contingent circle flash block,” the circle flashed when participants pressed the key in a half of the trials (flash trials), whereas the circle did not flash in the other half (no-flash trials). Moreover, because the flashing object attracts attention to the object^22,23^, we also examined the relationship between attention and the SoA^24,25^. We used “random circle flash block,” in which the circle flashed at random independently of keypresses in a half of the trials, and did not flash in the other half.

If task-irrelevant object responses enhanced the SoA for controlling (i.e., stopping) an object, the SoA rating would be higher in the flash trials of the action-contingent circle flash block. If attentional capture caused the high SoA, the SoA rating would also be higher for flash trials in the random circle flash blocks.

### Method

#### Participants

Participants were 24 volunteers (Age: 19–23 years; 10 women) with a normal or corrected-to-normal vision. They were naïve about the purpose of this study. We determined the sample size by a power analysis (α = 0.05, power = 0.80) using G*power 3.1^26^ for testing the medium effect size (f = 0.25). This sample size was similar to that in Nakashima and Kumada^21^ examining the SoA during automation (n = 27). All the experiments of this study were approved by Riken’s institutional review board. All participants gave their written informed consent before participating in the study. All experiments were performed in accordance with the relevant guidelines and regulations.

#### Apparatus, stimuli, and task

Presenting stimuli and recording the participants’ responses were controlled by a PC with Matlab software and Psychtoolbox^27,28^. We presented the stimuli on a 27-inch liquid crystal display (1920 × 1080 pixels, 60 Hz). Participants responded by using a standard 10-key-pad located in front of them. Participants were asked to press the response key using their preferred hand.

The background display (55.5° × 33.0° of visual angle) was a gray field with randomly distributed small white dots, in order to convey an illusion of friction. A grey square (named the goal, 150 × 150 pixels, 4.7° × 4.7°) was presented on the right side of the display. The center of the square was 1620 pixels (47.9°) from the left edge of the display. We told participants that they controlled (i.e., applied a brake to) a black circle with a diameter of 100 pixels (3.1°), which moved directly to the center of the display from the left side on each trial. The circle’s starting position was 100 pixels left from the left edge of the display such that participants could not see the circle when the trial started. The circle’s velocity varied from 600 to 880 pixels/sec in 20 pixels/sec increments (i.e., 15 velocities; 18.7–27.1°/sec) to reduce the monotony of the task.

We instructed participants to press the key once in each trial to apply a break to the circle. When they applied brakes, the circle slowed smoothly at a constant deceleration (60 × 9.8 pixels/s^2^, 18.3°/s^2^) and stopped. They were requested to try to stop the circle within the goal area as precisely as possible. We explicitly informed them that the experimental blocks contained automatic control trials where the circle stopped independently of their keypress. In the automatic control trials, the circle stopped at a location randomly determined within ± 250 pixels (± 7.8°) from the center of the goal. However, we only informed the participants that the circle stopped near the goal area in automatic control trials. We requested the participants to press the key in every trial, because they would not feel any SoA at all if they did not conduct any action. To make participants press the key, we included manual control trials in the experimental block, such that the circle stopped due to the participants’ keypress (there were 90 automatic control trials and 15 manual control trials in one experimental block). In manual control trials, the brake was applied 100 ms after participants’ keypress.

In each trial, a response display appeared after the circle stopped. Participants indicated the subjective ratings of their SoA for stopping the circle, i.e., the extent to which they felt that they had stopped the circle by themselves, by typing a number ranging from 0 to 10 (a higher number indicated a more robust SoA).

#### Procedure

Participants were tested individually. They sat on a chair, and a chin rest fixed their head at a 57 cm viewing distance. After listening to an explanation about the general experimental procedure, they performed 15 practice trials (no-flash trials), in which the brake was applied immediately after their keypress. The purpose of practice trials was to familiarize participants with the responding method and how the circle stopped. Then, they completed two experimental blocks, whose order was counterbalanced among participants. In each block (105 trials), we mixed 90 automatic control trials and 15 manual control trials. The experiment consisted of an action-contingent circle flash block in which the black circle flashed by changing from black to white when participants pressed the key in half the automatic control trials (i.e., 45 trials), and a random circle flash block in which the black circle flashed randomly unrelated to the participants’ keypress in half the automatic control trials. The circle did not flash in the manual control trials of either block.

In each trial, after the circle stopped, participants provided an SoA rating for stopping the circle in the response display. In addition to recording the SoA ratings (the primary dependent measure), we also recorded the participants’ key pressing time, the brake applied time, the circle-stop time, and the location of the circle stop in all the trials, and the circle-flash time in flash trials. Each recorded time was the elapsed time from a trial’s onset to when the event occurred.

The trial terminated 1000 ms after the circle passed through the right edge of the display, such that participants could no longer see the circle on the display during manual control trials, or 1000 ms after the circle stopped on automatic control trials, even if they failed to press the key. In these cases, after a warning display to remind participants to press the key, the response display appeared.

#### Data analysis

First, trials in which participants did not press the key were omitted from the analysis. We focused on data of automatic control trials and calculated each participant’s mean SoA ratings in the conditions. Then, we conducted a two-way repeated-measure analysis of variance (ANOVA) with SoA ratings for stopping the circle as the dependent variable and flash (flash vs. no-flash) and block (action-contingent flash vs. random flash) as independent variables. If the interaction was significant, we conducted post-hoc analyses using the simple main effect test.

### Results and discussion

We omitted 1.2% of the trials in the action-contingent circle flash block and 1.5% in the random circle flash block. Table 1 summarizes Experiment 1’s results (n = 24). The SoA ratings were relatively higher in the manual control trials than in the automatic control trials. An ANOVA on SoA ratings in the automatic control trials (Table 1a) indicated nonsignificant main effects, *F*s < 1.28, *p*s > 0.26, η_p_^2^ < 0.053, but a significant interaction, *F*(1,23) = 10.70, *p* = 0.003, η_p_^2^ = 0.318. Post-hoc comparison revealed that the SoA ratings were not different in either block when the circle did not flash, *F*(1,23) = 0.24, *p* = 0.625, η_p_^2^ = 0.011. Critically, compared to the SoA in no-flash trials, the SoA ratings were higher for action-contingent flashes, 6.08 (flash) vs. 5.82 (no-flash), *F*(1,23) = 8.87, *p* = 0.007, η_p_^2^ = 0.278, and lower for random flashes, 5.54 (flash) vs. 5.91 (no-flash), *F*(1,23) = 6.62, *p* = 0.017, η_p_^2^ = 0.223. This indicated that task-irrelevant action-contingent object flashes enhanced the participants’ SoA for stopping the object. The random flash block’s result suggested that this effect was not merely based on attention capture by abrupt luminance changes. Experiment 1 demonstrated that participants felt they controlled (i.e., stopped) an object that was automatically controlled if the object responded to their action, even though its responses were unrelated to stopping the object.

**Table 1:** Results in experiment 1.

	No-flash	Flash	Manual control
**(a) SoA ratings**			
Action contingent	5.82 ± 0.26	6.08 ± 0.24	6.80 ± 0.22
Random	5.91 ± 0.32	5.54 ± 0.30	6.89 ± 0.23
**(b) Distance between the circle and goal (pixels)**			
Action contingent	125.54 ± 1.76	124.96 ± 1.48	152.16 ± 15.13
Random	122.53 ± 2.24	125.77 ± 2.27	169.01 ± 19.57
**(c) Keypress timing from trial onset (ms)**			
Action contingent	1599 ± 38	1609 ± 38	1576 ± 40
Random	1604 ± 40	1608 ± 41	1586 ± 48
**(d) Imaginary circle stop position if the brake was applied by the participants’ keypress (pixels, Note: the left side of the display = 0 pixels)**			
Action contingent	1604 ± 28	1609 ± 27	1623 ± 32
Random	1607 ± 29	1610 ± 30	1601 ± 35

Mean ± SE. The values in Manual control are shown as reference information and not analyzed.

One possible explanation of Experiment 1’s results of SoA ratings in automatic control trials is that the performance of stopping the circle was different between the flash and no-flash trials. Studies suggested that performance judgments influence the SoA^29,30^. Moreover, SoA for continuously controlling an object increases with apparently better task performance through the intervention of supporting systems^18–20^. To investigate this possibility, we compared task performance, defined as the distance between the centers of the goal and the circle after stopping (i.e., error), among the conditions in automatic control trials (Table 1b). An ANOVA revealed neither main effects nor interaction, *F*s < 1, *p* > 0.379, η_p_^2^ < 0.034, indicating that performance did not differ among conditions. The task performance could not explain the results. The error values in this experiment were about 125 pixels. The circle stop position was randomly determined within ± 250 pixels from the center of the goal. Therefore, the mean error value should be 250/2 pixels in the automatic control trials, confirming that the automatic control system worked normally. It is noted that the error values were larger in the manual control trials, where the keypress reflected on the brake soon, than the automatic control trials. This is not incompatible with the suggestion that performance influences especially when the action-outcome relationship is uncertain^18,20^.

Another possible explanation of Experiment 1’s results is that participants’ actions differed between the flash conditions in automatic control trials. If they accidentally pressed the key later in action-contingent flash trials, the temporal delay between their action and the outcome might become shorter. Several studies have suggested that a short temporal delay between an action and an outcome leads to the higher SoA^31–35^. Thus, we compared the participants’ keypress timing among conditions (Table 1c). An ANOVA revealed neither main effects nor interaction, *F*s < 1.06, *p* > 0.315, η_p_^2^ < 0.044, indicating that the participants’ actions did not differ among conditions. The participants’ action could not explain the results.

We also calculated the imaginary circle stop position if the participants’ keypress stopped the circle in automatic control trials (Table 1d). We regarded participants whose mean circle stop position was over 300 pixels away from the goal in at least one condition as non-serious participants (5 participants), because they might not have followed the instruction to stop the circle near the goal. Even if we analyzed the SoA data of the remaining 19 participants, we obtained a similar significant interaction; *F*(1,18) = 5.68, *p* = 0.028, η_p_^2^ = 0.240; 6.17 (flash) vs. 5.97 (no-flash) in the action-contingent flash block, *p* = 0.046; 5.73 (flash) vs. 6.09 (no-flash) in the random flash block, *p* = 0.051.

Experiment 1 suggested that operators have a more robust SoA for controlling an object if the object responds to the operators’ action in automatic control situations, which we cannot explain by mere attentional capture for the object response. This indicates the crucial role of action-contingent responses. Participants in this experiment should feel SoA for flashing the object, because the object actually flashed with their keypress. The SoA resulting from one of the object’s responses to the operator’s actions might influence the SoA for the object’s other outcomes.

## Experiment 2

Experiment 1 demonstrated that action-contingent object responses enhanced the SoA during automation even if the response was irrelevant to control. In addition, the timing of the object response could be critical for optimum effect. That is, temporally-action-contingent object responses can enhance the SoA during automation.

In Experiment 2, we examined spatial characteristics of the object response effect. In one block of Experiment 2, identical to Experiment 1, the object responded to the participants’ action of pressing a key, i.e., the circle itself flashing, in a half of the trials. In the other block, another object responded to the participants’ action of pressing the key such that a white square abruptly appeared above the circle (Fig. 1c). In both blocks, an abrupt event occurs due to the participants’ actions in a half of the trials. Comparing the results of these two conditions would clarify whether the object’s response itself was essential or an event occurring in association with the participants’ action was critical for enhancing SoA during automation. If the object response were essential, the SoA would increase more when the circle responded to the action than when the square responded. Alternatively, if the mere occurrence of an associated event were adequate, the SoA would equally increase regardless of the condition.

### Method

Participants were new 24 volunteers (age: 18–24 years; 11 women) with a normal or corrected-to-normal vision. They were naïve about the purpose of this study. The apparatus, stimuli, task, and data analysis were identical to those in Experiment 1, except that we conducted two blocks; the circle flash block and another object (square) flash block. The procedure in the circle flash block was identical to the action-contingent circle flash block in Experiment 1. In the square flash block, a white square (150 × 150 pixels; 4.7° × 4.7°) suddenly and momentarily appeared just above the circle when participants pressed the key in a half of the automatic control trials. The distance between the centers of the square and the circle was 150 pixels. Participants rated the SoA for stopping the circle in each trial in both blocks.

### Results and discussion

We omitted 2.1% of the trials in the circle flash block and 2.1% in the square flash block from the analysis. Table 2 summarizes Experiment 2’s results (n = 24). The SoA ratings were relatively higher in the manual control trials than in the automatic control trials (Table 2a). The ANOVA indicated the significant interaction, *F*(1,23) = 4.38, *p* = 0.048, η_p_^2^ = 0.160. The post-hoc comparison revealed that the SoA ratings did not differ when the circle did not flash, *F*(1,23) = 0.37, *p* = 0.548, η_p_^2^ = 0.016. Again, the SoA rating were higher for circle flashes, 5.51 (flash) vs. 50.7 (no-flash), *F*(1,23) = 9.48, *p* = 0.005, η_p_^2^ = 0.292. Importantly, although the SoA rating appeared to be higher for the square flash, this difference was not significant, 5.16 (flash) vs. 4.97 (no-flash), *F*(1,23) = 1.87, *p* = 0.185, η_p_^2^ = 0.075. The main effect of flash was significant, *F*(1,23) = 6.14, *p* = 0.021, η_p_^2^ = 0.211, but the main effect of block was not, *F*(1,23) = 2.92, *p* = 0.101, η_p_^2^ = 0.112. We compared the errors (Table 2b) and keypress timings (Table 2c) among conditions. The results indicated neither main effects nor interactions for error, *F*s < 1, *p* > 0.379, η_p_^2^ < 0.034, and for keypress timing, *F*s < 1.80, *p* > 0.192, η_p_^2^ < 0.073. We confirmed that the system worked normally and that participants acted similarly in the two blocks. Again, when we analyzed the SoA data of the 20 serious participants based on the imaginary circle stop position (cf. Table 2d), we obtained similar results indicating a significant interaction, *F*(1,19) = 4.50, *p* = 0.047, η_p_^2^ = 0.191; 5.72 (flash) vs. 5.32 (no-flash) in the circle flash block; *p* = 0.026, 5.45 (flash) vs. 5.35 (no-flash) in the square flash block, *p* = 0.501.

**Table 2:** Results in experiment 2.

	No-flash	Flash	Manual control
**(a) SoA ratings**			
Circle flash	5.07 ± 0.25	5.51 ± 0.26	6.47 ± 0.23
Square flash	4.97 ± 0.26	5.16 ± 0.25	6.25 ± 0.26
**(b) Distance between the circle and the goal (pixels)**			
Circle flash	126.01 ± 2.39	127.38 ± 2.08	152.66 ± 8.67
Square flash	125.67 ± 2.66	128.02 ± 2.09	149.63 ± 8.47
**(c) Keypress timing from the trial onset (ms)**			
Circle flash	1753 ± 36	1750 ± 31	1731 ± 40
Square flash	1765 ± 35	1781 ± 33	1769 ± 28
**(d) Imaginary circle stop position if the brake was applied by the participants’ keypress (pixels, note: the left side of the display = 0 pixels, the goal position = 1620 pixels)**			
Circle flash	1716 ± 26	1714 ± 23	1682 ± 25
Square flash	1726 ± 25	1734 ± 25	1704 ± 16

Mean ± SE. The values in Manual control are shown as reference information and not analyzed.

The results of Experiment 2 suggest that the response of the object itself, rather than an event occurring in association with the participants’ action, was critical for enhancing the SoA for controlling the automatically controlled object. Experiments 1 and 2 demonstrated that spatiotemporally associated events with the participants’ actions (i.e., object response) change the participants’ feeling that they controlled the object.

## Follow-up analysis of experiments 1 and 2

**Figure 2:**
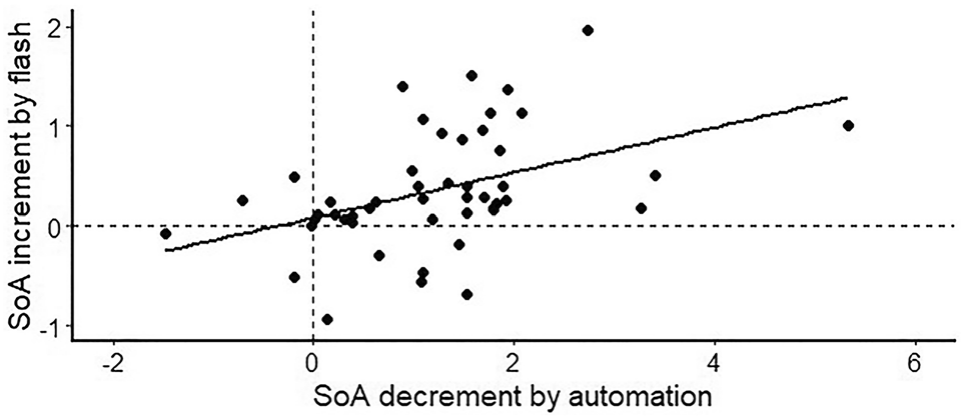
The correlation between SoA decrement by automation and SoA increment by the flash. Each dot indicates the data of one participant. The black line indicates the result of linear regression.

There could be large individual differences in the SoA because the SoA is a subjective experience. We investigated individual differences in the effect of the action-contingent object flash, although this was not this study’s primary purpose. Specifically, we examined the relationship between the SoA decrement by automation and the SoA increment by the flash during automation, using data of participants who performed the same task in the action-contingent object flash condition of Experiments 1 and 2 (n = 48). We defined the SoA decrement by automation as the value of subtracting automatic control trials’ mean rating (no-flash condition) from manual control trials’ mean rating, and the SoA increment by the flash as the value of subtracting the no-flash condition’s mean rating from the action-contingent flash condition’s mean rating in automatic control trials. There was a significant correlation between these two values, *r* = 0.431, *p* = 0.002 (Fig. 2).

The significant positive correlation indicates that action-contingent object responses more strongly increased the SoA of participants who felt less SoA during automation. Therefore, task-irrelevant object responses to action might be an effective way for increase the SoA of people who feel less involved in the control loop during automation. Future studies are necessary to investigate this issue in more detail.

## Experiment 3

Experiments 1 and 2 demonstrated that object responses spatiotemporally associated with participants’ actions enhanced the SoA for controlling the object during automation. Although Experiments 1 and 2 examined the SoA in automatic control situations only, action-contingent object responses might usually influence the SoA. Experiments 1 and 2 showed that the SoA ratings were lower in the automatic control trials, where the action was not related to the outcome, than in the manual control trials, where the action was reflected to the outcome immediately. Thus, the effect of the object response on SoA may be robust when it is difficult to associate an action with its outcome (i.e., the SoA becomes low) in manual control situations.

In Experiment 3, we conducted a task similar to Experiments 1 and 2 with one critical difference; participants informed that the circle would always stop due to their keypress (i.e., manual control situation), in order to examine whether action-contingent object response influenced the SoA for controlling an object in manual control situations. We manipulated the delay from the keypress to applying the brake to vary the uncertainty between a keypress and stopping the circle. The SoA decreases with longer delays between an action and its outcome^31–35^, suggesting that it is more difficult to recognize the action-outcome relationship with longer delays. Therefore, if the action-contingent object response effect were not limited to the SoA in automatic control situations, the object flash would increase the SoA rating with long delays in manual control situations. In contrast, if the object response effect is specific to automatic control situations, the SoA would not differ based on the object flash.

### Method

Participants were new 24 volunteers (Age: 19–23 years; 11 women) with normal or corrected-to-normal vision who were naïve about this study’s purpose. The apparatus, stimuli, and the task were identical to those in Experiment 1, except that all trials were manual control trials, and participants were informed about this. We manipulated the delay from the keypress to applying the brakes, 100, 300, or 500 ms. We conducted only the action-contingent circle flash block, which included 90 trials; 15 trials in each 3 (delay) × 2 (flash) conditions.

In the data analysis, we omitted trials in which participants pressed the key too late to stop the circle within the display from the analysis, because they could not see the circle stopping before rating their SoA. An ANOVA was applied to the SoA ratings with two factors: flash (flash vs. no-flash) and delay (100 vs. 300 vs. 500 ms). If the main effect of delay was significant, we conducted the post-hoc comparison by Holm’s Sequentially Rejective Bonferroni Procedure. In addition, an ANOVA was applied to the errors with flash and delay factors, although this was not our primary interest.

### Results and discussion

We omitted trials 9.9% of the trials from the analysis. Table 3a shows the SoA ratings in Experiment 3 (n = 24). An ANOVA revealed the significant main effect of the delay, *F*(2,46) = 73.13, *p* < 0.001, η_p_^2^ = 0.761, suggesting that the SoA decreased with longer delays, *p*s < 0.001. However, neither the main effect of the flash nor the interaction was significant, *F*s < 1, *p* > 0.598, η_p_^2^ < 0.023. There were 4 participants whose omitted data from analysis were above 20% of the trials. These participants might not have performed the task appropriately because they could not see the circle stopping in relatively many trials. The ANOVA indicated similar results even if we analyzed the data of the remaining 20 participants; a significant main effect of the delay, *F*(2,38) = 53.13, *p* < 0.001, η_p_^2^ = 0.736, no-significant other effects, *F*s < 1, *p* > 0.443, η_p_^2^ < 0.042.

**Table 3:** Results in experiment 3.

	No-flash	Flash
**(a) SoA ratings**		
100 ms delay	6.44 ± 0.26	6.30 ± 0.27
300 ms delay	5.55 ± 0.20	5.56 ± 0.22
500 ms delay	4.11 ± 0.27	4.18 ± 0.24
**(b) Distance between the circle and the goal (pixels)**		
100 ms delay	148.07 ± 11.29	146.57 ± 11.24
300 ms delay	138.73 ± 8.63	121.79 ± 6.45
500 ms delay	183.42 ± 6.86	184.39 ± 8.18

Mean ± SE.

We analyzed the errors (Table 3b). The error values were larger in the 500 ms delay condition than in other delay conditions, *F*(2,46) = 12.61, *p* < 0.001, η_p_^2^ = 0.354, indicating that the manual control task might be more difficult with longer delays. However, neither the main effect of the flash nor the interaction was significant, *F*s < 2.48, *p* > 0.129, η_p_^2^ < 0.098.

Longer delays induced lower SoA by making the relationship between the action and its effect uncertain, as suggested by previous studies^31–35^. However, there was no significant flash effect in Experiment 3, indicating that an object flash based on an action does not always influence the SoA for controlling the object. In addition, the differences of the SoA ratings between the flash and no-flash conditions were very small compared to those in the automatic control trials (i.e., Experiments 1 and 2). Therefore, the effect of the flash might be specific to automatic control situations, or at least to situations in which observers recognize the possibility that an automation system controls an object.

## General discussion

This study examined the effect of a task-irrelevant object response, an object flash, on the sense of agency (SoA) for stopping the object during an automatic control situation where the operator did not control the object. The results indicated that, firstly, the SoA for controlling the object increased when the object responded to an operators’ action. Secondly, spatiotemporally contingent object response to an operators’ action was critical for increasing the SoA. It is noted that the temporally contingent responses should be especially critical. The responses of a controlled object spatially associated with the object but temporally dissociated from the action significantly decreased the SoA (Experiment 1). In contrast, other object responses spatially dissociated from the controlled object but temporally associated with the action do not significantly increase/decrease the SoA (Experiment 2). Thirdly, task-irrelevant object responses influenced the SoA only in automatic control situations and not in manual control situations. Additionally, the effect of task-irrelevant object response on the SoA was more prominent for operators who felt less SoA when an automatic system controlled the object.

The participants’ action (i.e., keypress) in the automatic control trials of this study was not related to the outcome (i.e., the circle stopping). In contrast, the circle flash was associated with participants’ actions; therefore, the participants felt a robust SoA for the circle flash. The SoA for the circle flash might expand to stopping the circle, resulting in illusory feelings of SoA for system-derived outcome.

**Figure 3:**
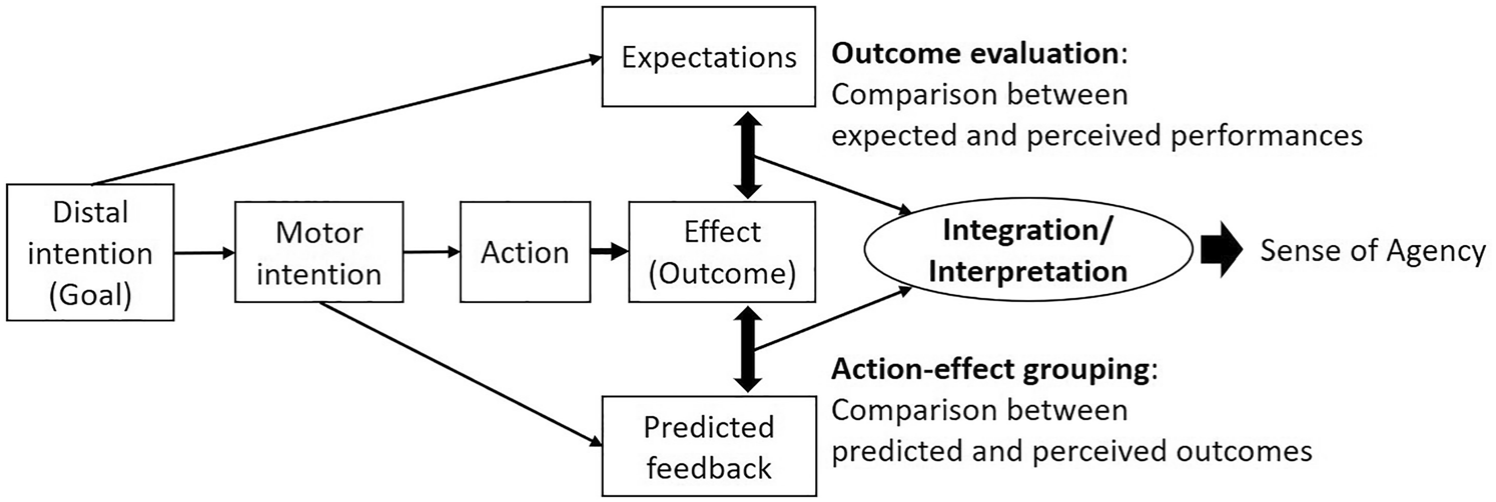
A simplified conceptual model of the sense of agency. Wen et al.^36^ described a more detailed model of the sense of agency. In this model, distal intentions produce performance expectations, and motor intentions produce predictions about feedback, which the model integrates by comparing them with actual action outcomes, thereby generating the SoA.

We discussed this study’s results (the SoA in the automatic control trials) based on the model of the SoA. The modified comparator model of SoA^36^ is a recent model explaining the SoA of people deciding on actions for achieving a specific goal (see Fig. 3 for a simplified conceptual model). This model provides an integrated description of predictive and postdictive processes in the SoA. In this model, the distal goal achievement and motor intentions produce two different pathways for comparison. First, expectations are generated by the distal intention and compared with the perception of performance in the outcome evaluation process. Second, similar to the original comparator model^37–40^, predictions of feedback are generated by motor intentions and compared with actual sensory feedback in the action-effect grouping process. Finally, integration of the two comparisons and interpretations generate the SoA.

In the modified comparator model^37^, the first pathway for modulating the SoA is outcome evaluation (performance evaluation), which refers to increasing SoA under good task performance, such that the SoA increases even if good task performance resulted from the system’s assistance^18–20^. Our results, however, showed that the task performance did not differ between flash and no-flash conditions. Therefore, we could not indicate that performance changes enhance the SoA in this study.

The second pathway is the action-effect grouping, which refers to the increasing SoA when action and an effect are spatiotemporally consistent. However, our results showed that the timing of action (participants’ keypress) was not different between flash and no-flash conditions, which did not indicate that the SoA increased because of accidental changes in participants’ action in the object flash condition (i.e., accidentally shortening the delay between the action and its outcome). Moreover, participants could not change their actions based on the object flash because the flash was associated with the action. Therefore, we did not expect that the duration between the action and outcome decreased in the object flash condition.

One might argue that task-irrelevant object responses synchronized the outcome (i.e., stop) resulting in a robust action-effect grouping (i.e., the perceived duration between the action and outcome becomes shorter). If that were the case, the object response should also increase the SoA in manual control trials. Nevertheless, Experiment 3 showed no SoA increment by the action-contingent flash during the manual control situation. Moreover, Haggard and Cole^41^ suggested that the intentional binding effect (the compressed perception of the interval between actions and outcomes^42^) is more robust when attention is divided between events than when it is focused on one event. Thus, counterintuitively, focusing attention on an object, possibly through attentional capture by action-contingent object responses, could make observers perceive that the duration between the action and the outcome is longer. In sum, we postulate that changes in action-effect grouping in response to object flashes are insufficient to explain SoA modulations in this study.

Because the results of this study cannot be explained by the modulation of the SoA model’s two pathways, object responses might influence the integration and interpretation process. Specifically, prior knowledge about the context of controlling an object, i.e., knowledge about manual or automatic control situations, might be crucial. Prior knowledge could modulate the judgement criterion of assignment of the outcome: Operators may judge the assignment of the outcome based on the criterion “by the observers themselves or not” in the manual control situation or the criterion “by the system or not” in the automatic control situation. Action-contingent object responses might make operators feel that the system controls the object less strongly during automatic control situations, which leads to the illusorily higher SoA. That is, by the object response, they tend to interpret that the outcome is not attributed to the system, and thus attributed to themselves. In contrast, when operators recognize that they control the object in manual control situations, action-contingent object responses might have little or no impact on the SoA because they basically feel that they control it by themselves. Farrer et al.^43^ suggested that, when the delay between an action and its effect becomes longer, participants feel that their action reflects the effect biasedly rather than others’ actions reflect the effect. Therefore, the lower SoA observed with longer delays might reflect participants’ realization that the effect does not directly reflect their action. In sum, the object response would not influence the SoA during manual control situations, if action-contingent object responses influence the outcome assignment criterion by making the criterion move closer to being “not by the system.”

This study’s experimental task required participants to behave goal-directedly, which might have caused the apparent purpose-sharing between the automatic control system and humans^21^. In addition, the system’s responses might have made participants feel that the system understands and mediates with their intention to accomplish the distal goal. These factors could enhance the reliability of the systems. These factors may create a new “we” identity between humans and systems similar to joint-action with other humans, resulting in a “we-agency” between them. Although previous studies have reported difficulty in producing a “we-agency” between humans and machines^44^, our study implies how the “we-agency” might develop during human–machine interaction.

There are some limitations to this study. Firstly, we only assessed subjective ratings of SoA. However, we could have also used other measurements to assess the SoA during automation effectively. For example, it would be necessary and interesting to examine whether visual processing or motivation is changed by illusory SoA during automation, because these could be modulated by the SoA^6–14^. Secondly, we should be cautious about generalizing our results to real-life situations. The enhancement of SoA by the object response is not useful when operators merely observe a controlling system (e.g., a supervisory task). The findings of this study are applicable only to situations where humans and systems operate jointly or humans partially control systems. Additionally, our experimental task required participants to act in every trial, and thus it is unclear whether the study’s findings can be applied to situations where operators are required to act infrequently. It is also necessary to examine our findings can be expand to the situation operators are required to act continuously^18–20^. There could be other approaches to maintaining the SoA in complete automations. Investigating such situations is essential for clarifying the details of the SoA during automation.

There are positive and negative aspects for maintaining the SoA during automation. Modern automation requires joint control by an operator and the system. Usually, the more predominant the automation system becomes in the operation, the less SoA the operator feels. The lack of the SoA might cause operators’ disengagement, which can be problematic when operators’ decision-making is required, namely “out-of-the loop” performance problem. Preventing this problem is a positive aspect of maintaining SoA in automatic control situations. In addition, the SoA enhancement may relate to building reliable human–machine interactions. On the other hand, the negative aspect of maintaining SoA during automation includes illusory SoA without actual control, which might cause problems in control behavior (e.g., careless behaviors) and cause dangerous results (e.g., accidents). Humans’ (subjective) responsibility for an operation should also be considered during automation when there is ambiguity about whether an operation is controlled by humans or machines (systems)^45,46^. Further, illusory SoA may be related to the ethics in system design, where the automation system designers may deceive users/operators. In order to comprehensively address the SoA during automation, it is necessary to consider both the positive and negative aspects of maintaining/enhancing the SoA from the broader perspective. Many issues remain to be investigated in this field. We believe that the results of this study provide specific clues on the relevance of SoA in automation technology^47^.

In conclusion, this study suggests that control-irrelevant object response to an operators’ actions enhances the SoA for controlling objects during automation. The spatiotemporal contingency between the operators’ action and the object response is crucial for increasing the SoA. These suggestions are important for developing collaborative operator-automation systems and ensuring efficient joint control by such systems.

## Data Availability

The datasets analyzed during the current study are available at the Open Science Framework, URL: https://osf.io/5smuz/.
